# Variation in Enamel Formation Genes Influences Enamel Demineralization *In Vitro* in a *Streptococcus mutans* Biofilm Model

**DOI:** 10.3389/fphys.2017.00851

**Published:** 2017-10-30

**Authors:** Liangyue Pang, Qinghui Zhi, Peilin Zhuang, Lixia Yu, Ye Tao, Huancai Lin

**Affiliations:** ^1^Guangdong Provincial Key Laboratory of Stomatology, Sun Yat-Sen University, Guangzhou, China; ^2^Department of Preventive Dentistry, Guanghua School of Stomatology, Sun Yat-Sen University, Guangzhou, China; ^3^Department of Stomatology, Sun Yat-Sen Memorial Hospital, Sun Yat-Sen University, Guangzhou, China

**Keywords:** dental caries, dental enamel, enamel demineralization, enamel formation genes, polymorphism

## Abstract

Genetic studies have shown that variations in enamel formation genes are associated with caries susceptibility. The aim of this study was to test *in vitro* whether variants in these genes are associated with dental enamel demineralization in a *Streptococcus mutans* biofilm model. DNA and enamel samples were obtained from 213 individuals. DNA was extracted from saliva, and 16 single nucleotide polymorphisms were analyzed. The physical and chemical properties of sound enamel samples and the mineral loss and the lesion depth of the demineralized enamel samples under cariogenic challenge were analyzed. Microhardness, enamel chemicals, mineral loss and demineralization depth were compared between different genotypes at each single nucleotide polymorphism. The GG genotype of *TUFT1* (rs17640579) and the GT genotype of *MMP20* (rs1612069) exhibited increased microhardness (*p* = 0.044 and 0.016, respectively). The GG genotype of *AMBN* (rs7694409) had a higher magnesium level, while the CT genotype of *TFIP11* (rs2097470) had a lower magnesium level (*p* = 0.044 and 0.046, respectively). The GT genotype of *MMP20* (rs1612069) had a higher calcium level (*p* = 0.034). The GG genotype of *AMBN* (rs13115627), the AG genotype of *ENAM* (rs12640848) and the AA genotype of *MMP20* (rs2292730) had a lower phosphorus level (*p* = 0.012, 0.006, and 0.023, respectively). The GG genotype of *AMBN* (rs13115627) was also associated with a higher calcium-phosphorus ratio (*p* = 0.034). Individuals with the CC genotype of *TFIP11* (rs134143) exhibited significantly more mineral loss (*p* = 0.011) and a deeper lesions (*p* = 0.042). Individuals with the TT genotype of *TFIP11* (rs2097470) had more mineral loss (*p* = 0.018). Individuals with the GG genotype of *TUFT1* (rs17640579) exhibited a shallower demineralization depth (*p* = 0.047). Individuals with the GT genotype of *MMP20* (rs1612069) exhibited a shallower demineralization depth (*p* = 0.042). Individuals with the GG genotype of *ENAM* (rs12640848) exhibited less mineral loss (*p* = 0.01) and a shallower demineralization depth (*p* = 0.03). Genetic variations in *TFIP11, TUFT1, MMP20*, and *ENAM* influenced enamel demineralization in a *Streptococcus mutans* biofilm model.

## Introduction

Enamel is the most highly mineralized tissue in the human body. The amelogenesis phase of enamel development is strictly controlled by enamel formation genes (Fincham et al., 1999), and the size, shape, structure and composition of enamel are affected by genetic variations (Simmer and Hu, 2001). Dental caries is initiated by the demineralization of enamel, which results from the production of acid from sugar by a plaque biofilm. Genetic association studies of dental caries have suggested that caries may be influenced by variations in enamel formation genes, such as ameloblastin (*AMBN*), amelogenin (*AMELX*), enamelin (*ENAM*), matrix metalloproteinase 20 (*MMP20*), tuftelin (*TUFT1*), and tuftelin-interacting protein 11 (*TFIP11*) (Shimizu et al., 2012; Wang et al., 2012; Gasse et al., 2013; Shaffer et al., 2015; Gerreth et al., 2016). Previous studies have sought to explain how enamel formation genes influence caries susceptibility (Shimizu et al., 2012; Daubert et al., 2016; Uhlen et al., 2016). One hypothesis is that variation in these genes results in the formation of enamel that is more susceptible to cariogenic challenge (Shimizu et al., 2012).

The effects of enamel formation genes on caries susceptibility are impacted by environmental factors, such as fluoride exposure (Shaffer et al., 2015). *In vitro* investigations allow the relationships between variations in enamel formation genes and enamel vulnerability to be investigated in isolation. Using an *in vitro* pH-cycling model, a previous study demonstrated that genetic variations in enamel formation genes are associated with changes in enamel microhardness (Shimizu et al., 2012). However, direct exposure to acid does not simulate the bacterial biofilm interactions that characterize caries formation *in vivo*. In addition, previous work showed that *Streptococcus mutans* (*S. mutans*) appears to interact with genetic variations in enamel formation genes to influence caries susceptibility (Slayton et al., 2005).

*S. mutans* plays a decisive role in the development of dental caries, and its existence influences the effects of enamel formation genes on caries susceptibility (Slayton et al., 2005). Thus, a study aimed at determining the influence of variations in enamel formation genes on enamel demineralization under cariogenic challenge in a *S. mutans* biofilm model would provide meaningful information to the field. To the best of our knowledge, no previous study has explored this issue. The aim of this study was to investigate the relationships between variations in enamel formation genes and both the loss of minerals and the depth of enamel demineralization *in vitro* in a *S. mutans* biofilm model. We hypothesized that genetic variations in these genes influence enamel vulnerability, resulting in more or less mineral loss and deeper or shallower lesion depth under artificial cariogenic challenge.

## Materials and methods

### Calculation of study sample size

Single nucleotide polymorphisms (SNPs) (Table 1) were selected from HapMap {www.hapmap.org [CHB database, HapMap release 27 (2009, February)]} using Haploview 4.2. We were particularly interested in *AMELX* (rs946252), *AMBN* (rs4694075), *ENAM* (rs12640848), *TUFT1* (rs3790506), and *MMP20* (rs1784418), which have been reported to be associated with caries susceptibility (Slayton et al., 2005; Shimizu et al., 2012; Tannure et al., 2012; Gerreth et al., 2016). The minor allele frequencies (MAFs) of these SNPs in the Han Chinese population are shown in Table 1. The sample size was calculated according to the MAF of rs12640848, which was the lowest among the SNPs of interest. A one-way design with multiple levels was applied, and the formula used to calculate the sample capacities is shown below.

$$\begin{array}{l} N=\frac{\left(q_{1}^{-1}+q_{2}^{-1}\right){\left(Z_{\frac{\alpha }{2}}+Z_{\beta }\right)}^{2}S^{2}}{\delta^{2}} \end{array}$$

The value of α was set at 0.017, and the value of β was set at 0.1. The values of *q*_1_ and *q*_2_ indicate the proportions of the different genotypes. When calculating the sample size for microhardness, δ and *S* were used to represent the *D*-value between the microhardness among the different genotypes and the standard deviation of enamel microhardness within the same genotype. When calculating the sample size for the enamel chemicals, δ and *S* were used to represent the *D*-value between the phosphorus level of the enamel among the different genotypes and the standard deviation of the phosphorus level within the same genotype. When calculating the sample size for enamel demineralization after a carious challenge, δ and *S* were used to represent the *D*-value between the demineralization depth under cariogenic challenge among the different genotypes and the standard deviation of the demineralization depths within the same genotype. Before formal experiments were performed, 10 enamel blocks that were obtained from individuals with different genotypes of rs12640848 were used to measure the microhardness, phosphorus level and demineralization depth following an artificial caries challenge. The average microhardness of the AA, AG, and GG genotypes were 315.43, 325.03, and 341.60, respectively, with standard deviations of 12.17, 14.95, and 12.95, respectively. Therefore, a sample size of 122 individuals was necessary to analyze the relationship between the enamel formation gene polymorphisms and microhardness. The average enamel phosphorus level of the AA, AG, and GG genotypes were 18.75 Wt%, 18.09 Wt%, and 18.43 Wt%, respectively, with standard deviations of 0.13, 0.21, and 0.27, respectively. As a result, a sample size of 116 individuals was necessary to analyze the relationship between the enamel formation gene polymorphisms and the enamel chemicals. For the AA, AG, and GG genotypes, the average demineralization depths were 195.2, 200.4, and 187.2 μm, respectively, with standard deviations of 6.76, 10.47, and 8.42, respectively. Based on this information, a sample size of 209 individuals was necessary to analyze the relationship between the enamel formation gene polymorphisms and enamel demineralization.

**Table 1:** Candidate genetic markers evaluated in this study.

**Gene name**	**Locus**	**Marker public ID**	**Base pair exchange (MAF)**
AMELX	Xp22.31 – p22.1	rs946252	C/T (0.427)
AMBN	4q21	rs4694075	C/T (0.464)
		rs7694409	A/G (0.244)
		rs13115627	A/G (0.250)
ENAM	4q13.3	rs12640848	A/G (0.292)
TUFT1	1q21	rs6587597	G/A (0.399)
		rs16833391	C/T (0.161)
		rs17640579	A/G (0.321)
		rs12749	G/A (0.232)
		rs3790506	G/A (0.321)
TFIP11	22q12.1	rs2097470	C/T (0.286)
		rs134143	T/C (0.363)
			
KLK4	19q13.41	rs198968	A/G (0.131)
MMP20	11q22.3-q23	rs2292730	G/A (0.202)
		rs1784418	C/T (0.470)
		rs1612069	G/T (0.429)

### Selection of premolars and saliva samples

**Figure 1:**
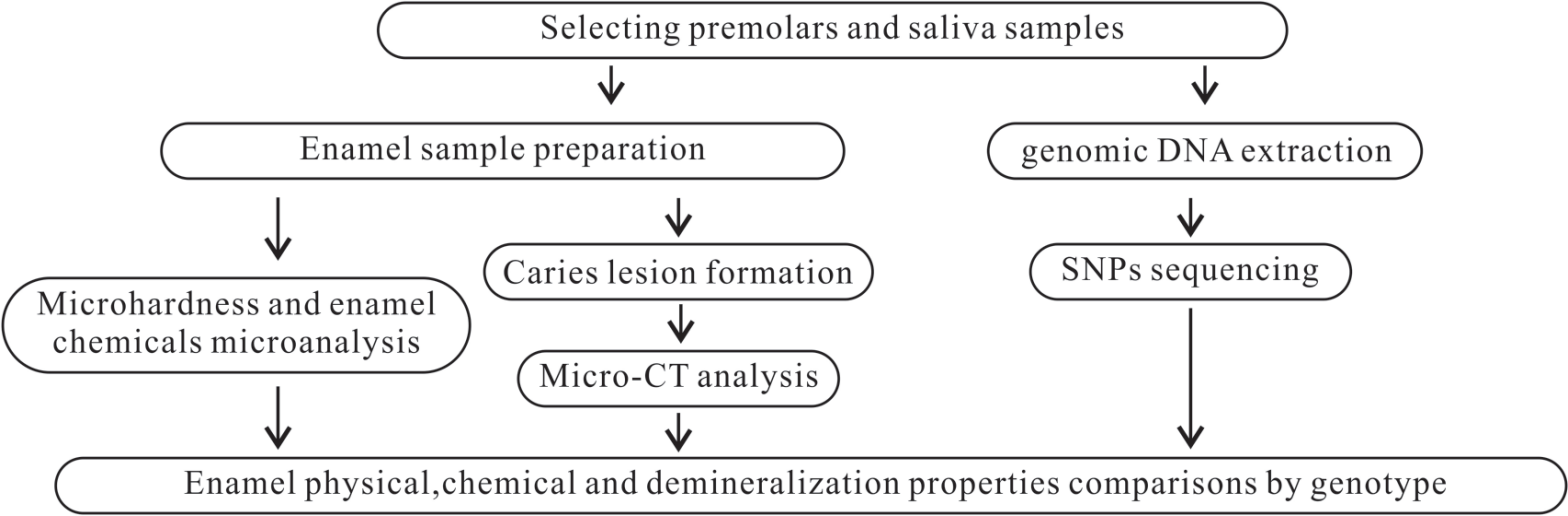
Summary of study design.

The project was approved by the Ethical Review Committee of Guanghua School of Stomatology, Sun Yat-Sen University (ERC-2015-15). The subjects were given verbal and written information regarding the study, and they provided written consent to participate. Eligible unrelated individuals were recruited using quota sampling at the Department of Oral and Maxillofacial Surgery, Hospital of Stomatology, Sun Yat-Sen University and Guangdong Provincial Stomatological Hospital from September 2015 to January 2017. These hospitals are the two largest stomatological hospitals in Guangdong Province. To be eligible for inclusion in the study, subjects were required to be the following: (1) of Chinese Han ethnicity, (2) aged between 13 and 18 years old, (3) individuals whose premolars were extracted for orthodontic reasons, and (4) systemically healthy. Furthermore, teeth were required to be/have the following: (1) complete development of the apical part of the root, (2) a sound premolar without visible caries or restorations, and (3) healthy teeth without enamel hypoplasia or dental fluorosis. One to three premolars from each subject were collected and stored in a container containing 0.1% thymol solution at 4°C until use. Saliva samples (2 ml each) were obtained from each participant and stored using Oragene DNA Self-Collection kits (Lang Fu, China). In total, 213 subjects were enrolled in this study. Those subjects contributed 447 teeth. Figure 1 summarizes the study design.

### Enamel sample preparation

**Figure 2:**
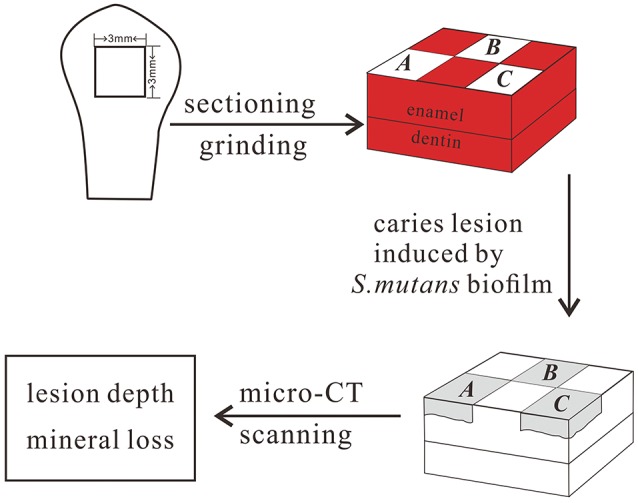
Preparation of enamel specimens.

Before each tooth was sectioned, it was thoroughly cleaned of debris and gingival tissues. The teeth were cut in half mesially–distally and coronally–apically, leaving the buccal surface halves. The lingual halves and the root were removed using a cutter bar (Accutom-50, Struers, Denmark). The flattest central portion of the buccal surface was used to prepare a 3 × 3 × 3 mm cuboidal tooth block. One enamel specimen was prepared from each tooth. The prepared enamel block was polished on a rotating polishing machine using progressively finer grades of SiC grinding paper under water cooling (Tegramin preparation system, Struers, Denmark). It was previous reported that when the outer 100 μm of the enamel is polished, there is no significant difference in superficial microhardness between unerupted enamel and enamel 10 years after eruption (Palti et al., 2008). In our study, the upper 150–200 μm of surface enamel, measured with a Vernier caliper, was polished to avoid the influence of post-eruptive maturation on the results. Among those enamel samples, 134 enamel blocks (one from each participant) were used to test the correlation between enamel microhardness and genetic variations in the enamel formation gene markers, and 130 enamel blocks (one from each participant) were used to test the correlation between the enamel chemicals and genetic variations in the gene markers described above. Furthermore, 213 enamel blocks (one from each participant) were used to carry out the artificial caries challenge and to test the correlation between enamel demineralization and genetic variation in the enamel formation gene markers. For the artificial caries challenge, each specimen contained a horizontal 3 × 3 mm enamel surface, which was divided into 6 windows of 1.5 × 1 mm each. All of the surfaces of the specimens except those shown in windows A, B, and C (Figure 2) were coated with acid-resistant nail varnish (Miniso, Guangzhou, China).

### Microhardness analysis

The microhardness of each specimen was determined by a microhardness tester (DuraScan-20; Struers, Denmark) at a load of 100 g for 15 s. The average surface microhardness was determined from three indentations placed in the center of the surface of each specimen. The spaced indentations were 100 μm away from each other.

### Enamel chemicals microanalysis

Elemental microanalyses were performed using an Electron Probe Micro Analyzer (JXA-8800R, JEOL, Tokyo, Japan) equipped with Wavelength Dispersive X-ray Spectroscopy (WDS). The standards used for calibration were Apatite [Ca5 (PO4)5(F, Cl, OH)], for calcium and phosphate, and diopside (MgO) for magnesium. The counting time at each point was 10 s with a 1 mm diameter of the electron beam at 15.0 kV and 20 nA. In this test, the relative amounts of the three elements and the calcium-phosphorus ratio were calculated based on the mean of the three points which were measured in the center of the surface of each specimen. Each point was approximately 50 μm away from each other.

### Induction of caries lesion formation by *S. mutans* biofilm

The tooth blocks were subjected to steam autoclaving (121°C for 15 min) (Amaecha et al., 1999) and placed individually into the wells of a 24-well plate (Corning, NY, USA). To induce acquired pellicle biofilm formation, the tooth blocks were pre-conditioned with sterile artificial saliva (Leagene, Beijing, China) at 37°C (Wei et al., 2011). After 2 h, all of the tooth blocks were transferred to a new 24-well plate, and each well was inoculated with 0.1 ml *S. mutans* (1 × 10^7^ CFU/mL) and 1.9 ml 1% BHIS. The *S. mutans* used in this study was UA159, which was provided by Guangdong Institute of Microbiology. The enamel specimens were incubated under anaerobic conditions at 37°C for 6 h to allow initial bacterial adhesion to the saliva-coated blocks. The enamel blocks were then transferred to fresh 1% BHIS and incubated overnight under anaerobic conditions at 37°C to allow the biofilm to mature (Cavalcanti et al., 2014). Then, the specimens were exposed to 10% sucrose for 5 min three times per day to simulate cariogenic challenge. The cariogenic challenges were performed at predetermined times (8:00 a.m., 12:30 a.m., and 5:00 p.m.). After each cariogenic challenge, the blocks were washed three times in 0.9% NaCl and returned to the original medium. The culture medium was changed twice daily after the first and last sucrose exposure (Giacaman et al., 2015). The experimental phase lasted 10 days to allow the formation of artificial caries lesions. Following caries lesion formation, the specimens were rinsed with deionized water for 2 min to dislodge the attached biofilm. All the laboratory manipulations that involved *S. mutans* were performed in class II biosafety cabinets. The 24-well plate used in the study, the experimental waste and the enamel blocks were subjected to steam autoclaving (121°C for 15 min).

### Micro-CT scanning and analysis

After artificial caries formation, each tooth block was placed in a resin tube 9 mm in diameter and scanned using micro-CT (μCT 50, Scanco Medical AG, Switzerland). The X-ray source was operated at a voltage of 70 kV with a current of 200 kA and an exposure time of 1,500 ms. The highest spatial resolution used during scanning was 5 μm. A 0.5-mm aluminum filter was used to block the weakest X-rays. During scanning, the tooth block was placed in the tube with the exposed enamel surface perpendicular to the sidewall of the tube. Then, a wet sponge was placed in the tube to fix the position of the tooth block and maintain block moisture. The scanning results for each specimen were reconstructed using μCT-reconstruction software (μCT50, Scanco, Bassersdorf, Switzerland). The reconstructed 3D images were viewed and processed using μCT evaluation software (μCT50, Scanco, Bassersdorf, Switzerland). From the reconstructed 3D images of each specimen, cross-sectional images of each specimen were located. From these lesion images, 10 were randomly selected. Lesion depth was measured using the image analysis software Image J (National Institutes of Health, USA) (Mei et al., 2013). To calibrate mineral density, a series of mineral reference phantoms (Phantoms, Scanco Medical AG, Switzerland) were scanned prior to specimen scanning. Four phantoms (100, 200, 400, and 800 mgHA/cm^3^) were scanned using the same setup and parameters as those used to scan the tooth specimens. The gray values of 10 points were measured in the images selected for each HA phantom. They were then averaged and plotted against the mineral density value of the phantoms to calculate the following calibration equation:

$$\begin{array}{l} \text{Mineral density}=\left(\text{Gray level value}\times 0.1834\right)-35.778. \end{array}$$

This calibration equation was used to transform the gray level values of the images into true mineral density (MD) values. The MD value of the region of interest (ROI) in the 10 selected cross-sectional images was measured. In the carious lesions, the ROI was 20 × 20 pixels (100 × 100 μm) located precisely in the middle of the lesion just below the exposed tooth surface. In the healthy part, the ROI was 20 × 20 pixels (100 × 100 μm) in the corresponding position, which was located by determining the same distance from the upper surface. Measurements were taken in each lesion and protected area, and the mean of these measurements was used as the MD of the lesion and sound enamel. Mineral loss (%) was calculated by subtracting the lesion MD value from the sound enamel MD value and then dividing the result by the sound enamel MD value (Lo et al., 2010; Neves et al., 2016).

### DNA analysis

Genomic DNA was extracted from saliva samples according to the manufacturer's instructions. Spectrophotometry was used to determine the DNA concentration and purity of each sample. The DNA concentration was evaluated at 260 nm, and the ratio of the readings obtained at 260 and 280 nm was used to estimate DNA purity. Sixteen markers in seven genes known to be involved in enamel formation were selected for analysis in this study. The rs134143 genotype was determined by direct sequencing and genotyping of other 15 SNPs was performed using matrix-assisted laser desorption/ionization time-of-flight mass spectrometry (MALDI-TOF MS).

### Statistical analysis

All of the data were analyzed on a personal computer using SPSS 22.0. Before analysis, the mineral loss data were transformed using the arcsine square root transformation. Because neither mineral loss nor lesion depth had a normal distribution, a general linear model was used to investigate the influence of SNP status on enamel microhardness, chemicals and enamel demineralization properties. Intergroup comparisons were performed using a *post hoc* analysis with the LSD test. The relationship between sex and either the enamel physical and chemical properties or enamel demineralization was analyzed using a *t*-test. The level of statistical significance was set at 0.05.

## Results

### Study population

Two hundred and thirteen subjects (155 females and 58 males) were included in the study. Sixteen SNPs distributed across the following seven enamel matrix genes were investigated: *AMELX, AMBN, ENAM, TFIP11, TUFT1*, KLK4, and *MMP20* (Table 1). All of the genotypes were in Hardy–Weinberg equilibrium. The mean microhardness of the initial enamel was 337.35 (±21.63). The mean magnesium, calcium, and phosphorus contents and the calcium-phosphorus ratio were 0.18 Wt % (±0.05), 39.26 Wt % (±0.58), 18.53 Wt % (±0.35), and 2.12 (±0.07) respectively. The mean lesion depth was 195.48 μm (±10.94), and the mean mineral loss was 49.08% (±5.4%). No significant interaction was found between sex and either the enamel properties or enamel demineralization (Tables 2–4, respectively).

**Table 2:** Enamel microhardness by sex.

**Sex**	**Hardness Mean ± *SD***	***p*-value**
Male (*n* = 58) Female (*n* = 155)	339.54 ± 23.19 336.17 ± 20.79	0.408^*^

* *p-value of the comparisons of the microhardness by sex using a t-test*.

**Table 3:** Enamel chemical properties by sex.

**Sex**	**Magnesium level (Wt %) Mean ± *SD***	***p*-value**	**Calcium level (Wt %) Mean ± *SD***	***p*-value**	**Phosphorus level (Wt %) Mean ± *SD***	***p*-value**	**Calcium-phosphorus radio Mean ± *SD***	***p*-value**
Male (*n* = 41) Female (*n* = 89)	0.19 ± 0.04 0.18 ± 0.05	0.386^*^	39.36 ± 0.92 39.21 ± 0.88	0.391^*^	18.56 ± 0.34 18.51 ± 0.34	0.467^*^	2.12 ± 0.08 2.12 ± 0.07	0.847^*^

* *p-value of the comparisons of the enamel chemicals by sex using a t-test*.

**Table 4:** Enamel demineralization properties by sex.

**Sex**	**Lesion depth (μm) Mean ± *SD***	***p*-value**	**Mineral loss (%) Mean ± *SD***	***p*-value**
Male (*n* = 58) Female (*n* = 155)	194.57 ± 10.75 196.87 ± 10.91	0.14^*^	49.15 ± 5.2 49.25 ± 5.7	0.90^*^

* *p-value of the comparisons of the demineralization properties by sex using a t-test*.

### Enamel physical and chemical properties analysis

Table 5 demonstrates the association between SNP status and the surface microhardness. The GT genotype at rs1612069 of *MMP20* and the GG genotype at rs17640579 of *TUFT1* were associated with increased enamel hardness (*p* = 0.016 and 0.044, respectively). Table 6 demonstrates the association between SNP status and the enamel chemicals. The GG genotype at rs7694409 of *AMBN* had a higher magnesium level (*p* = 0.044), while the CT genotype at rs2097470 of *TFIP11* was associated with a lower magnesium level (*p* = 0.046). The GT genotype at rs1612069 of *MMP20* had a higher calcium level (*p* = 0.034). The GG genotype at rs13115627 of *AMBN*, the AG genotype at rs12640848 of *ENAM* and the AA genotype at rs2292730 of *MMP20* were associated with a lower phosphorus level (*p* = 0.012, 0.006 and 0.023, respectively). The GG genotype at rs13115627 of *AMBN* also had a higher calcium-phosphorus ratio (*p* = 0.034).

**Table 5:** Summary of the microhardness comparisons by genotype.

**Gene**	**Gene marker**	**Genotype (n)**	**Micro-hardness of the enamel Mean ± *SD***	***p*-value**
		CC (52)	336.35 ± 26.59	
AMELX	rs946252	CT (45)	337.42 ± 19.00	0.943^#^
		TT (36)	337.72 ± 16.55	
		CC (22)	337.29 ± 22.04	
AMBN	rs4694075	CT (62)	336.35 ± 24.21	0.861^#^
		TT (50)	338.62 ± 18.14	
		AA (89)	337.05 ± 20.27	
	rs7694409	AG (36)	338.31 ± 25.25	0.618^#^
		GG (6)	328.89 ± 20.76	
		AA (89)	337.67 ± 20.31	
	rs13115627	AG (37)	337.25 ± 25.16	0.917^#^
		GG (8)	334.33 ± 20.93	
		AA (79)	336.66 ± 23.68	
ENAM	rs12640848	AG (44)	338.03 ± 19.50	0.887^#^
		GG (11)	339.61 ± 14.34	
		AA (62)	336.38 ± 21.33	
KLK4	rs198968	AG (62)	338.17 ± 23.05	0.891^#^
		GG (10)	338.30 ± 14.86	
		CC (80)	336.48 ± 19.98	
TFIP11	rs2097470	CT (46) TT (8)	340.62 ± 20.55 327.33 ± 38.47	0.236^#^
		CC (6)	336.67 ± 23.03	
	rs134143	CT (65)	340.73 ± 22.67	0.206^#^
		TT (63)	333.93 ± 20.16	
		AA (8)	339.96 ± 17.84	
MMP20	rs2292730	AG (51)	336.55 ± 23.49	0.907^#^
		GG (75)	337.62 ± 20.90	
		CC (39)	333.54 ± 23.76	
	rs1784418	CT (62)	339.50 ± 18.78	0.403^#^
		TT (33)	337.84 ± 23.99	
		GG (37)	336.24 ± 21.96	
	rs1612069	GT (67)	341.18 ± 19.34	**0.016**^#^
		TT (28)	327.49 ± 22.74	
		AA (25)	344.56 ± 15.78	
TUFT1	rs6587597	AG (70)	336.79 ± 21.57	0.236^#^
		GG (38)	333.06 ± 24.20	
		CC (96)	335.85 ± 20.62	
	rs16833391	CT (34)	341.48 ± 23.46	0.365^#^
		TT (3)	330.11 ± 32.17	
		AA (64)	334.55 ± 20.86	
	rs17640579	AG (55)	337.16 ± 22.96	**0.044**^#^
		GG (15)	350.00 ± 15.73	
		AA (8)	324.46 ± 38.61	
	rs12749	AG (42)	341.03 ± 18.46	0.127^#^
		GG (84)	336.74 ± 20.80	
		AA (14)	346.90 ± 21.14	
	rs3790506	AG (61)	337.72 ± 21.67	0.162^#^
		GG (58)	337.33 ± 21.58	

# *p-value of the comparisons of the microhardness by genotype using a general linear model*.

*The significant P-values of the microhardness comparisons by genotype have been highlighted in bold*.

**Table 6:** Summary of the enamel chemical comparisons by genotype.

**Gene**	**Gene marker**	**Genotype (n)**	**Magnesium content (Wt %) Mean ± *SD***	***p*-value**	**Calcium content (Wt %) Mean ± *SD***	***p*-value**	**Phosphorus content (Wt %) Mean ± *SD***	***p*-value**	**calcium-phosphorus ratio Mean ± *SD***	***p*-value**
		CC (51)	0.18 ± 0.05		39.30 ± 0.94		18.51 ± 0.33		2.12 ± 0.07	
AMELX	rs946252	CT (40)	0.19 ± 0.04	0.361^#^	39.33 ± 0.90	0.633^#^	18.51 ± 0.36	0.599^#^	2.13 ± 0.07	0.471^#^
		TT (37)	0.18 ± 0.05		39.15 ± 0.84		18.58 ± 0.37		2.11 ± 0.07	
		CC (21)	0.19 ± 0.05		39.36 ± 0.83		18.39 ± 0.43		2.14 ± 0.07	
AMBN	rs4694075	CT (62)	0.18 ± 0.05	0.479^#^	39.14 ± 0.89	0.330^#^	18.59 ± 0.36	0.073^#^	2.11 ± 0.07	0.093^#^
		TT (47)	0.18 ± 0.03		39.38 ± 0.92		18.51 ± 0.28		2.13 ± 0.07	
		AA (77)	0.18 ± 0.04		39.35 ± 0.93		18.51 ± 0.32		2.13 ± 0.07	
	rs7694409	AG (44)	0.18 ± 0.05	**0.044**^#^	39.21 ± 0.85	0.418^#^	18.58 ± 0.34	0.151^#^	2.11 ± 0.07	0.513^#^
		GG (6)	0.22 ± 0.05		38.90 ± 0.84		18.30 ± 0.69		2.13 ± 0.12	
		AA (78)	0.18 ± 0.04		39.32 ± 0.93		18.50 ± 0.32		2.13 ± 0.07	
	rs13115627	AG (45)	0.18 ± 0.05	0.061^#^	39.13 ± 0.79	0.415^#^	18.62 ±0.34	**0.012**^#^	2.10 ± 0.06	**0.034**^#^
		GG (7)	0.22 ± 0.05		39.49 ± 1.19		18.22 ± 0.56		2.17 ± 0.11	
		AA (81)	0.18 ± 0.04		39.26 ± 0.87		18.60 ± 0.33		2.11 ± 0.07	
ENAM	rs12640848	AG (38)	0.18 ± 0.05	0.590^#^	39.20 ± 0.90	0.769^#^	18.39 ± 0.37	**0.006**^#^	2.13 ± 0.07	0.222^#^
		GG (11)	0.19 ± 0.05		39.43 ± 1.08		18.47 ± 0.29		2.14 ± 0.07	
		AA (53)	0.18 ± 0.05		39.34 ± 0.92		18.45 ± 0.39		2.13 ± 0.08	
KLK4	rs198968	AG (64)	0.18 ± 0.04	0.260^#^	39.19 ± 0.88	0.641^#^	18.58 ± 0.31	0.103^#^	2.11 ± 0.06	0.174^#^
		GG (13)	0.16 ± 0.04		39.30 ± 0.89		18.57 ± 0.36		2.12 ± 0.07	
		CC (75)	0.19 ± 0.04		39.29 ± 0.83		18.57 ± 0.31		2.12 ± 0.06	
TFIP11	rs2097470	CT (49)	0.17 ± 0.04	**0.046**^#^	39.25 ± 0.98	0.792^#^	18.47 ± 0.41	0.300^#^	2.13 ± 0.08	0.663^#^
		TT (6)	0.18 ± 0.07		39.03 ± 1.05		18.54 ± 0.37		2.11 ± 0.08	
		CC (4)	0.20 ± 0.08		39.00 ± 1.36		18.64 ± 0.38		2.09 ± 0.10	
	rs134143	CT (67)	0.17 ± 0.04	0.075^#^	39.27 ± 0.90	0.841^#^	18.46 ± 0.40	0.077^#^	2.13 ± 0.08	0.334^#^
		TT (59)	0.19 ± 0.04		39.27 ± 0.87		18.60 ± 0.28		2.11 ± 0.06	
		AA (11)	0.16 ± 0.04		39.47 ± 0.80		18.29 ± 0.34		2.16 ± 0.07	
MMP20	rs2292730	AG (43)	0.18 ± 0.04	0.340^#^	39.37 ± 0.94	0.375^#^	18.49 ± 0.33	**0.023**^#^	2.13 ± 0.07	0.050^#^
		GG (75)	0.18 ± 0.05		39.17 ± 0.88		18.58 ± 0.35		2.11 ± 0.07	
		CC (43)	0.18 ± 0.03		39.26 ± 0.96		18.50 ± 0.36		2.12 ± 0.08	
	rs1784418	CT (62)	0.19 ± 0.05	0.066^#^	39.28 ± 0.86	0.944^#^	18.57 ± 0.37	0.291^#^	2.12 ± 0.07	0.807^#^
		TT (25)	0.17 ± 0.05		39.21 ± 0.87		18.45 ± 0.28		2.13 ± 0.06	
		GG (37)	0.17 ± 0.04		39.07 ± 0.86		18.56 ± 0.35		2.11 ± 0.07	
	rs1612069	GT (70)	0.19 ± 0.05	0.152^#^	39.44 ± 0.88	**0.034**^#^	18.54 ± 0.37	0.469^#^	2.13 ± 0.07	0.265^#^
		TT (21)	0.18 ± 0.03		38.97 ± 0.94		18.45 ± 0.30		2.11 ± 0.07	
		CC (95)	0.18 ± 0.05		39.31 ± 0.92		18.54 ± 0.32		2.12 ± 0.07	
TUFT1	rs16833391	CT (32)	0.17 ± 0.03	0.216^#^	39.14 ± 0.82	0.638^#^	18.47 ± 0.45	0.452^#^	2.12 ± 0.08	0.938^#^
		TT (2)	0.19 ± 0.01		39.40 ± 1.16		18.74 ± 0.03		2.10 ± 0.07	
		AA (21)	0.18 ± 0.05		39.12 ± 0.79		18.59 ± 0.38		2.11 ± 0.07	
	rs6587597	AG (70)	0.18 ± 0.04	0.848^#^	39.20 ± 0.91	0.324^#^	18.53 ± 0.34	0.461^#^	2.12 ± 0.07	0.231^#^
		GG (38)	0.18 ± 0.04		39.43 ± 0.91		18.48 ± 0.36		2.14 ± 0.07	
		AA (60)	0.18 ± 0.04		39.33 ± 0.89		18.47 ± 0.35		2.13 ± 0.07	
	rs17640579	AG (54)	0.18 ± 0.05	0.893^#^	39.20 ± 0.93	0.739^#^	18.55 ± 0.36	0.127^#^	2.11 ± 0.07	0.282^#^
		GG (15)	0.19 ± 0.06		39.23 ± 0.84		18.67 ± 0.33		2.10 ± 0.07	
		AA (5)	0.16 ± 0.05		39.12 ± 0.86		18.49 ± 0.29		2.12 ± 0.06	
	rs12749	AG (44)	0.17 ± 0.04	0.184^#^	39.26 ± 0.80	0.931^#^	18.49 ± 0.41	0.652^#^	2.12 ± 0.07	0.880^#^
		GG (80)	0.19 ± 0.05		39.28 ± 0.95		18.55 ± 0.35		2.12 ± 0.07	
		AA (12)	0.17 ± 0.05		39.29 ± 0.85		18.43 ± 0.46		2.13 ± 0.08	
	rs3790506	AG (63)	0.18 ± 0.05	0.521^#^	39.21 ± 0.88	0.790^#^	18.57 ± 0.33	0.382^#^	2.11 ± 0.07	0.481^#^
		GG (54)	0.18 ± 0.04		39.32 ± 0.93		18.50 ± 0.36		2.13 ± 0.07	

# *p-value of the comparisons of the enamel chemical by genotype using a general linear model*.

*The significant P-values of the enamel chemical comparisons by genotype have been highlighted in bold*.

### Enamel demineralization analysis

**Figure 3:**
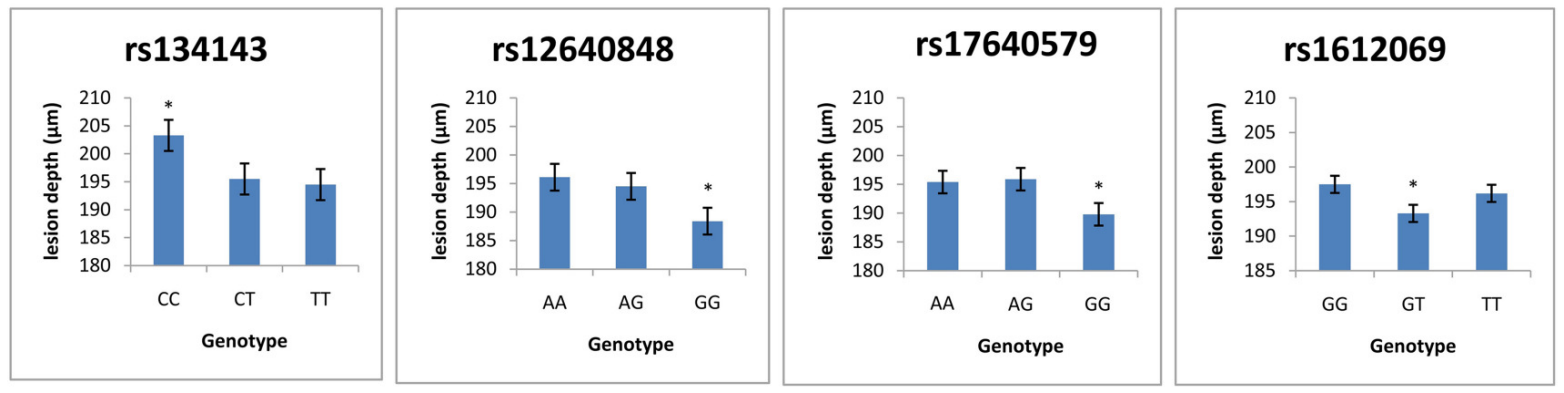
Comparison of enamel lesion depth by genotype. ^*^Significant difference from the wild genotype.

**Figure 4:**
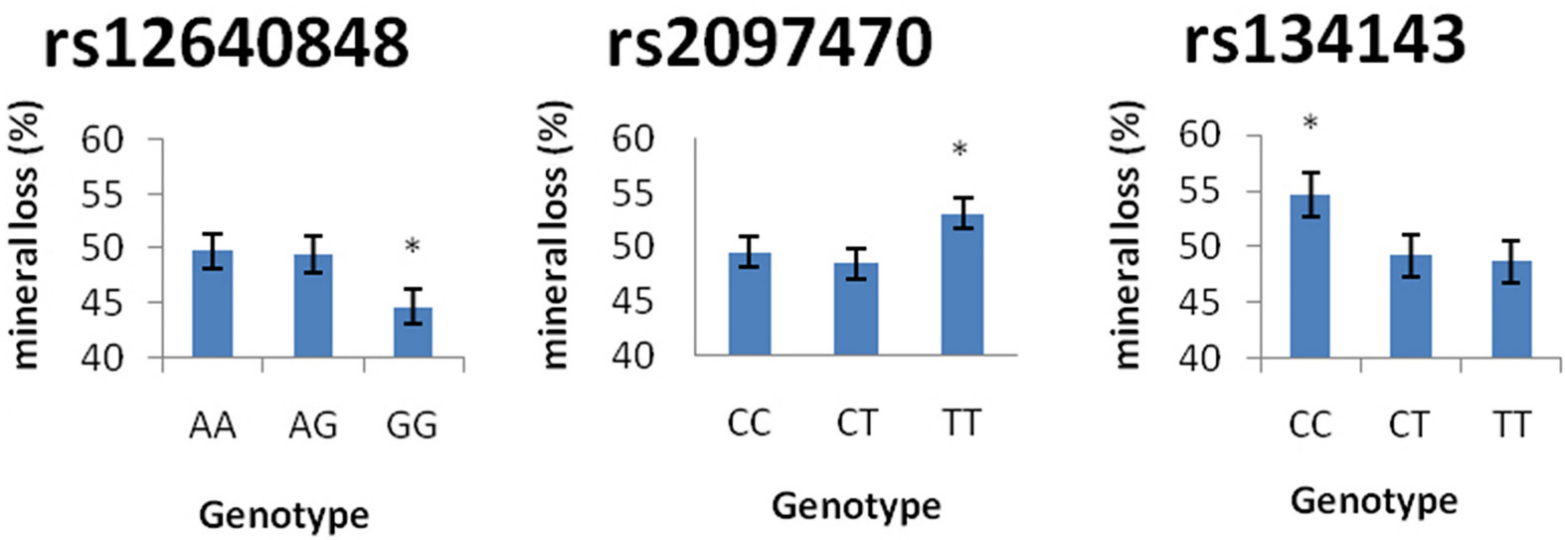
Comparison of enamel mineral loss by genotype. ^*^Significant difference from the wild genotype.

Table 7 demonstrates the association between SNP status and enamel mineral loss or lesion depth. Significant associations were identified between mineral loss in enamel and both rs2097470 (*p* = 0.018) and rs134143 of *TFIP11* (*p* = 0.011) and rs12640848 of *ENAM* (*p* = 0.01). Moreover, we discovered that four genetic variants, rs17640579 of *TUFT1* (*p* = 0.047), rs12640848 of *ENAM* (*p* = 0.03), rs134143 of *TFIP11* (*p* = 0.042) and rs1612069 of *MMP20* (*p* = 0.042), were significantly associated with the depth of enamel demineralization. The alternative CC genotype at rs134143 of *TFIP11* was associated with a higher degree of enamel loss and a thicker lesion depth, and the TT genotype at rs2097470 of *TFIP11* was associated with a higher degree of enamel loss. The GG genotype at rs12640848 of *ENAM* was associated with a lower degree of enamel loss and a shallower demineralization depth. The GG genotype at rs17640579 of *TUFT1*was associated with a shallower demineralization depth, and the GT genotype at rs1612069 of *MMP20* was associated with a thinner lesion depth (Figures 3, 4).

**Table 7:** Summary of the lesion depth and loss of mineral comparisons by genotype.

**Gene**	**Gene marker**	**Genotype (n)**	**Lesion depth (μm) Mean ± SD**	***p*-values**	**Mineral loss (%) Mean ± SD**	***p*-values**
		CC (83)	194.2 ± 9.6		49.13 ± 5.51	
AMELX	rs946252	CT (67)	195.1 ± 11.8	0.49^#^	49.87 ± 5.94	0.67^#^
		TT (60)	196.3 ± 10.4		49.10 ± 5.14	
		TT (76)	194.9 ± 10.1		50.26 ± 4.92	
AMBN	rs4694075	CT (102)	195.1 ± 11.1	0.99^#^	49.16 ± 6.02	0.16^#^
		CC (34)	195.2 ± 9.8		48.20 ± 5.03	
		AA (139)	195.7 ± 10.2		49.80 ± 5.24	
	rs7694409	AG (58)	193.6 ± 11.1	0.34^#^	48.32 ± 6.01	0.25^#^
		GG (10)	197.5 ± 10.1		49.20 ± 4.70	
		AA (140)	195.4 ± 10.4		49.80 ± 5.33	
	rs13115627	AG (61)	194.0 ± 10.8	0.64^#^	48.72 ± 5.99	0.30^#^
		GG (12)	195.7 ± 10.7		48.10 ± 4.88	
		AA (134)	195.9 ± 9.9		49.75 ± 5.89	
ENAM	rs12640848	AG (66)	194.5 ± 11.5	**0.03**^#^	49.45 ± 4.29	**0.01**^#^
		GG (12)	187.9 ± 7.9		44.83 ± 5.25	
		GG (16)	191.8 ± 7.9		49.38 ± 5.67	
KLK4	rs198968	AG (101)	195.3 ± 10.9	0.44^#^	49.18 ± 5.29	0.85^#^
		AA (96)	195.4 ± 10.4		49.63 ± 5.75	
		TT (13)	200.9 ± 12.5		53.15 ± 6.81	
TFIP11	rs2097470	CT (73)	194.8 ± 9.9	0.1^#^	48.48 ± 5.40	**0.018**^#^
		CC (126)	194.5 ± 10.5		49.50 ± 5.29	
		CC (11)	202.6 ± 13.1		54.27 ± 6.10	
	rs134143	CT (101)	195.1 ± 10.5	**0.042**^#^	49.14 ± 5.45	**0.011**^#^
		TT (100)	194.2 ± 10.1		49.12 ± 5.31	
		AA (17)	198.9 ± 10.3		49.47 ± 5.59	
MMP20	rs2292730	AG (72)	194.4 ± 9.7	0.283^#^	48.46 ± 5.66	0.22^#^
		GG (122)	195.0 ± 11.0		49.38 ± 5.52	
		CC (66)	194.3 ± 10.7		48.60 ± 5.30	
	rs1784418	CT (103)	195.3 ± 10.4	0.750^#^	49.73 ± 5.26	0.39^#^
		TT (44)	195.5 ± 10.6		49.80 ± 6.33	
		GG (58)	197.7 ± 10.0		49.05 ± 6.03	
	rs1612069	GT (109)	193.6 ± 9.6	**0.042**^#^	49.72 ± 5.11	0.62^#^
		TT (43)	196.1 ± 9.6		48.93 ± 5.81	
		GG (64)	194.9 ± 10.7		48.46 ± 5.27	
TUFT1	rs6587597	AG (112)	195.6 ± 9.9	0.53^#^	49.64 ± 5.63	0.21^#^
		AA (36)	193.4 ± 11.9		50.36 ± 5.46	
		CC (152)	194.9 ± 10.0		49.07 ± 5.48	
	rs16833391	CT (55)	195.1 ± 11.8	0.76^#^	50.02 ± 5.45	0.26^#^
		TT (5)	198.4 ± 11.3		52.40 ± 7.23	
		GG (21)	189.8 ± 12.1		48.52 ± 4.85	
	rs17640579	AG (89)	196.0 ± 9.7	**0.047**^#^	50.10 ± 5.82	0.29^#^
		AA (102)	195.4 ± 10.7		48.97 ± 5.36	
		GG (128)	195.1 ± 9.9		49.25 ± 5.50	
	rs12749	AG (73)	195.2 ± 11.3	0.96^#^	49.75 ± 5.57	0.78^#^
		AA (11)	194.3 ± 13.0		48.82 ± 5.82	
		GG (94)	196.0 ± 10.8		48.99 ± 5.79	
	rs3790506	AG (95)	194.9 ± 10.3	0.25^#^	49.80 ± 5.43	0.59^#^
		AA (23)	191.9 ± 10.1		49.30 ± 4.81	

# *p-value of the comparisons of the lesion depth and loss of mineral by genotype using a general linear model*.

*The significant P-values of the lesion depth and the loss of mineral comparisons by genotype have been highlighted in bold*.

## Discussion

Previous studies have shown that genetic variations in enamel formation genes influence caries risk (Opal et al., 2015). One plausible hypothesis of how those genes are associated with caries lesion development is via interactions with oral bacteria (i.e., *S. mutans*) (Wang et al., 2012). In our study, considering the importance of *S. mutans* in the initiation and development of caries, we used an *in vitro* biofilm model of *S. mutans* to investigate the relationship between enamel formation gene polymorphism and enamel demineralization. Although *S. mutans*-only biofilm was used to induce caries lesion formation in the current study, it is worthwhile to point out that a multispecies microbial biofilm model can create a more diverse microbial environment and better stimulate the intra-oral conditions associated with natural caries.

Tooth type, tooth surface (buccal or lingual) and post-eruptive enamel maturation are regarded as factors that influence enamel demineralization (Palti et al., 2008; Mistry et al., 2015). To avoid the influences of these potentially confounding factors on our results, only one surface of one type of tooth was used in the present study (the buccal surface of sound premolars that were extracted for orthodontic reasons), and 150–200 μm of the surface enamel was removed in a controlled manner using a Vernier caliper. Previous studies of the correlations between changes in acid-induced enamel microhardness or demineralization-associated lesions and genetic variations in enamel formation genes included small samples of 28 and 90 subjects (Shimizu et al., 2012; Uhlen et al., 2016). The present study included 213 individuals. To the best of our knowledge, the relationships between polymorphisms in enamel formation genes and enamel demineralization have never previously been studied in such a large population.

The demineralization of dental enamel can be quantified by evaluating changes in dental mineral content using a variety of techniques (Bergman and Engfeldt, 1954; Carlstroem, 1964). Transverse microradiography (TMR) is considered the gold standard technique for determining the mineral content of cariogenic lesions (Huysmans and Longbottom, 2004). However, this technique has disadvantages, including its inherently destructive nature and the fact that it is extremely difficult to prepare specimens for TMR. Micro-CT can be used to obtain 3D images without destroying the specimen and without the need to prepare thin slices (Arends and ten Bosch, 1992). A previous study showed that micro-CT scanning is as sensitive as TMR for detecting changes in mineral content (Lo et al., 2010). Therefore, we used micro-CT to analyse mineral loss and lesion depth in demineralized enamel in an artificial caries model.

Previous studies had shown that microhardness and enamel chemicals, such as calcium and magnesium were related to genetic variations in enamel formation genes (Shimizu et al., 2012; Halusic et al., 2014). Therefore, we investigated the physical and chemical properties of enamel as phenotypic features that would distinguish further genotypes for the various genes interrogated in this study. In the present study, we found variations in rs17640579 of *TUFT1* and rs1612069 of *MMP20* associated with microhardness. Shimizu et al had reported variations in those two genes associated with microhardness of the buccal surface at different SNPs (Shimizu et al., 2012). One possible explanation for these findings is that a SNP that is significantly associated with phenotypes is not necessarily the causative variant itself because it might be in a strong linkage disequilibrium with other true-positive SNPs that have not yet been identified in the gene region (Gerreth et al., 2017).

We also found significant associations between variations in *AMBN, ENAM, TFIP11, MMP20* and enamel chemicals. Our results suggested that GG genotype of *AMBN* rs7694409 associated with a higher magnesium level and GG genotype of *AMBN* rs13115627 associated with a lower phosphorus level and a higher calcium-phosphorus ratio. Halusic et al. previously proved that variations in *AMBN* rs4694075 contribute to a lower calcium levels in primary teeth (Halusic et al., 2014). Our results also indicated that variations in rs12640848 of *ENAM* were associated with a lower phosphorus level. However, Halusic et al. found variation in *ENAM* rs12640848 was associated with lower calcium and magnesium concentrations. Both a genome-wide association study and a laboratory study found that the genetic influences on enamel were different between primary and permanent dentitions (Wang et al., 2010; Shaffer et al., 2011; Bayram et al., 2015). Permanent teeth were used in the present study, which may explain our results. Meanwhile, compared to the research carried out by Halusic et al. which only included the calcium and magnesium concentrations, in the present study, we analyzed the relative amounts of calcium, phosphorus and magnesium in the enamel (Halusic et al., 2014). This difference may be another possible explanation for our results. Moreover, we also found that the CT genotype at rs2097470 of *TFIP11* was associated with lower magnesium level; the GT genotype in rs1612069 of *MMP20* had a higher calcium level and the AA genotype at rs2992730 of *MMP20* was associated with lower phosphorus level. Our results demonstrated that variations in enamel formation genes could possibly contribute to changes of the magnesium, calcium and phosphorus levels in enamel.

In our study, individuals with the GG genotype at rs12640848 of *ENAM* had less mineral loss and lower lesion depths than individuals with the AA and AG genotypes of rs12640848, indicating that this genotype might exert a protective effect against enamel demineralization. Shimizu et al. detected an association between genetic variation in rs12640848 of *ENAM* and changes in enamel microhardness at the distal surface but not the buccal surface, exposed to an acidic pH environment (Shimizu et al., 2012). In the present study, we induced cariogenic challenge using a *S. mutans* biofilm model rather than an acidic pH environment, and one possible explanation for our results is that *S. mutans* influences the relationship between genetic variation at SNP rs12640848 and enamel demineralization. Another possible explanation is that the index that was used to measure the demineralization lesion was different between the two studies. We assessed the mineral status of the demineralized lesion instead of assessing microhardness. Microhardness is used to measure mechanical properties and structural integrity and is not always strongly correlated with mineral loss when evaluating demineralization at lesion sites (Lippert and Lynch, 2014). Previous epidemiological surveys have also found that the GG genotype at rs12640848 exerts a protective effect against caries (Abbasoglu et al., 2015; Gerreth et al., 2016). The results of previous studies, in combination with our results, reinforce the notion that enamel formation genes contribute to the incidence of dental caries by altering the ability of dental enamel to resist cariogenic challenge.

Previous epidemiological studies have reported an association between caries susceptibility and genetic variations at *MMP20* rs1784418, *TUFT1* rs3790506 (Slayton et al., 2005; Shimizu et al., 2012; Filho et al., 2017). We found no association between genetic variations in these SNPs and enamel demineralization following cariogenic challenge. Instead, other SNPs in *MMP20* and *TUFT1*, including *MMP20* rs1612069 and *TUFT1* rs17640579, were associated with demineralization depth in our study. One possible explanation for these findings is that a SNP that is significantly associated with disease occurrence is not necessarily the causative variant because it might be linked to other true-positive SNPs that have not yet been identified in the gene that were passed down to the next generation (Gerreth et al., 2017). Environmental factors might also influence the effects of variations in these genes on caries susceptibility (Wang et al., 2012; Slade et al., 2013). The microhardness test also found the GT genotype of *MMP20* rs1612069 and the GG genotype of *TUFT1* rs17640579 led to harder enamel. Previous findings found that softer enamel may demineralize easier (Shimizu et al., 2012); therefore, it is easy to understand that individuals with the GT genotype in *MMP20* rs1612069 exhibit a shallower demineralization depth than individuals with GG genotype of rs1612069 and individuals with GG genotype in *TUFT1* rs17640579 exhibit a shallower demineralization depth than individuals with AA and AG genotype of rs17640579. Our data demonstrate that these genotypes might exert protective effects against enamel demineralization in response to cariogenic challenge in this study population.

Tuftelin-interacting protein 11 (*TFIP11*) has been identified as a protein that interacts with tuftelin (*TUFT1*), which has been suggested to play an important role during the development and mineralization of enamel. Significant associations were found between genetic variations in two SNPs of *TFIP11* and enamel demineralization in response to cariogenic challenge. Individuals with the CC genotype in *TFIP11* rs134143 exhibit deeper demineralization and more mineral loss. Individuals with the TT genotype in *TFIP11* rs2097470 exhibited more mineral loss than individuals with TT and CT genotype at rs2097470 in response to cariogenic challenge. These results suggest that the CC genotype in *TFIP11* rs134143 and TT genotype in *TFIP11* rs2097470 might be a risk factor for enamel demineralization.

In conclusion, we identified significant associations between genetic variations in *TFIP1, TUFT1, ENAM*, and *MMP20* and enamel demineralization using an *in vitro* biofilm model of *S. mutans*. Our findings demonstrate that variations in enamel formation genes influence enamel demineralization in response to cariogenic challenge. The results of the current study also provide support for the notion that genetic changes in enamel formation genes can lead to susceptibility or resistance to demineralization in response to cariogenic challenge and that these effects influence an individual's susceptibility to dental caries. The genes examined in our study are involved in different stages of the formation of tooth hard tissue, ranging from the formation of the enamel matrix to the mineralization and structural organization of the enamel (Vieira et al., 2014; Smith et al., 2017). Our data also found genetic variations in enamel formation genes associated with enamel microhardness and chemicals. Based on these results, we hypothesize that genetic variations in these genes may lead to abnormal protein functions or decreased amounts of protein, contributing to microstructural or component alterations in enamel that cause higher or lower levels of mineral to be lost in response to cariogenic challenge (Patir et al., 2008; Chaussain et al., 2014). Future research should focus on whether and how variations in genes involved in amelogenesis influence the protein functions or amounts of protein and how these variants interact with exposure to environmental factors.

## Author contributions

LP contributed to the study conception and design, data acquisition and analysis, and the drafting of the manuscript; YT, LY, and PZ contributed to the data acquisition; QZ contributed to the data analysis and drafting of the manuscript; and HL contributed to the study conception and design, data acquisition and analysis, and manuscript revisions. All authors read and critically revised the manuscript. All authors gave final approval and agreed to be accountable for all aspects of the work.

### Conflict of interest statement

The authors declare that the research was conducted in the absence of any commercial or financial relationships that could be construed as a potential conflict of interest.
